# Burnout, Organizational Justice, Workload, and Emotional Regulation among Medical and Non-Medical Personnel Working in Romanian Healthcare Units

**DOI:** 10.3390/bs13030225

**Published:** 2023-03-04

**Authors:** Roxana Mihaela Claponea, Magdalena Iorga

**Affiliations:** 1 Faculty of Psychology and Education Sciences, “Alexandru Ioan Cuza” University of Iasi, 700554 Iasi, Romania; 2 Behavioral Sciences Department, Faculty of Medicine, “Grigore T. Popa” University of Medicine and Pharmacy, 700115 Iasi, Romania

**Keywords:** medical staff, non-medical staff, burnout, emotional regulation, organizational justice, workload

## Abstract

Background and objectives: The goal of this study was to evaluate the levels of organizational justice, emotional regulation, and workload associated with the level of burnout experienced in medical and non-medical staff from public and private medical units. Materials and Methods: A cross-sectional study was conducted on a sample of 230 healthcare professionals, including 139 medical personnel and 91 non-medical staff respondents. The collected socio-demographic and organizational data and psychological tools were the Maslach Burnout Inventory (MBI HSS), the ECO System, and the emotional regulation questionnaire (ERQ). Results: For medical staff, burnout was measured in terms of emotional exhaustion (M = 27.05 ± 12.34), depersonalization (M = 8.26 ± 3.95), and personal accomplishment (M = 47.35 ± 6.78). The scores for non-medical staff were emotional exhaustion (M = 35.84 ± 14.71), depersonalization (M = 11.79 ± 6.30), and personal accomplishment (M = 44 ± 7.37). In terms of workload, higher scores were observed for non-medical staff (M = 25.43 ± 7.87), while medical staff recorded lower values (M = 20.35 ± 7.65). The scores for the cognitive reappraisal dimension were as follows: medical personnel (M = 32.02 ± 5.37) and non-medical staff (M = 31.67 ± 6.19). In terms of the expressive suppression dimension, medical staff registered at M = 17.99 ± 5.61, and non-medical staff registered at M = 17.19 ± 5.53. For organizational justice, higher scores were registered for medical staff (M = 25.87 ± 6.02) and lower scores for non-medical staff (M = 21.34 ± 5.72). Conclusions: Medical staff felt a higher sense of organizational justice than non-medical staff, as is also evidenced by the level of the workload dimension, which registers higher values for non-medical personnel. In the case of burnout, higher levels of emotional exhaustion and depersonalization dimensions were also revealed for non-medical staff and, in the case of the professional fulfillment dimension, higher scores were registered for medical staff.

## 1. Introduction

Burnout is a psychological response to prolonged periods of exposure to stress [1]. The concept of burnout was first introduced by Herbert Freudenberger in 1974, who studied burnout by observing increasingly exhausted co-workers displaying a lack of motivation at work. The author described burnout as a feeling of exhaustion arising from excessive efforts to achieve idealistic expectations, a feeling of physical and emotional exhaustion, or the result of failure resulting from unrealistic expectations, whether self- or socially imposed.

Burnout can make an individual feel ineffective, exhausted, and distant in relation to work, thus limiting their productivity and success [2]. It has three dimensions: emotional exhaustion, depersonalization, and personal accomplishment. Emotional exhaustion is the central emotional component of the syndrome that triggers the burnout process. This dimension indicates a depletion of emotional resources caused by sustained occupational stress. Depersonalization (cynicism) is the interpersonal dimension of burnout and indicates detachment in interpersonal relationships. Personal accomplishment is the self-evaluation dimension of burnout, which manifests itself in decreased self-esteem and self-worth. The continuous mismatch between work demands and capabilities leads to emotional exhaustion, accompanied by changes in attitude and behavior, namely, depersonalization and reduced personal accomplishment [3].

Organizational psychosocial factors that can lead to stress, burnout, or other health problems are represented by excessive demands, a lack of control over the demands of a particular job, a lack of support from colleagues and supervisors, poor working relationships and management, and physical or verbal violence [4]. The European Agency for Safety and Health at Work classifies psychosocial risks at work as excessive workload, conflicting demands, a lack of clarity about the worker’s role, a lack of involvement in decision-making and a lack of influence over how the work is carried out, poor management of organizational changes, job insecurity, ineffective communication, and a lack of support from management or colleagues, as well as psychological and sexual harassment and violence from third parties [5].

Workload becomes overload when the demands of the job exceed human limits [4]. This is one of the core factors facilitating the development of burnout, when it becomes a chronic work condition rather than an occasional emergency [6]. Quantitatively, workload looks at the number of tasks that need to be accomplished; qualitatively, it looks at the difficulty of the tasks as well as the information that needs to be processed [7]. Not infrequently, a high workload has been associated with deteriorating well-being and the occurrence of health problems [8]. Thus, individuals are constrained to perform multiple tasks with fewer resources at their disposal. Under these conditions, a high workload is correlated with the occurrence of burnout, mainly in the emotional exhaustion dimension [6].

The concept of organizational justice was popularized by Adams’ equity theory [9], and it is primarily associated with employees’ perceptions of fairness in an organization. In other words, the concept refers to employees’ perceptions of fairness. Organizational justice can lead to the emergence of emotional and physical health disturbances in health professionals and others. Through these disturbances, employees may feel that they are being treated unfairly at work or may feel a lack of reciprocity in terms of social interactions [10]. Organizational justice is important in any organization, but for healthcare organizations, it appears indispensable.

Medical staff, represented by nurses, doctors, pharmacists, physiotherapists, biologists, and psychologists, are characterized by demands with an increased degree of difficulty, responsibility, and strong commitment. Nurses, analyzed as an indispensable component of the healthcare workforce, spend most of their lives in healthcare facilities and constitute the largest professional group in the hospital [11,12]. The most common psychosocial risks for healthcare staff are a heavy workload, a lack of organizational justice, emotional regulation, or role conflicts [13].

The administrative personnel of a hospital play the role of providing and processing the various data needed in the hospital’s decision-making process [14]. Human Resources Management, Planning, Finance, and Accounting are all departments that play central roles in a hospital. They are responsible for fulfilling the vision and performance of the healthcare organization while ensuring that the hospital is developed harmoniously through the planning, execution, and auditing of specific hospital management activities. As such, the roles of administrative staff working in such departments are essential [14].

In a meta-analysis conducted by Aronsson et al. [15], focusing on the association between work conditions and the development of burnout symptoms in health professionals, it was highlighted that emotional demands were studied in relation to emotional exhaustion in several studies [16,17,18,19]. At the same time, a high workload has been associated with the emotional exhaustion dimension [17,19,20,21,22,23,24], as well as with depersonalization or cynicism [19,21,23,24]. Regarding the protective factor against burnout, organizational justice has been associated with emotional exhaustion [24,25,26]; thus, at a reduced level of organizational justice, a high level of emotional exhaustion is felt. Previous studies have correlated low levels of justice with burnout [19,21,23,24,25,26].

Shin et al. [27] investigated the relationship between coping strategies and burnout symptoms and showed that cognitive reappraisal was negatively correlated with burnout symptoms. Significant positive correlations were also reported between emotional regulation and burnout. Some aspects of emotional regulation, such as emotional suppression or attempts to identify one’s inner self with desired emotions, have been considered a kind of emotional experience common in nurses at work [28]. Although both strategies can be effective in increasing emotional expressive behavior, cognitive reappraisal reduces the experience of disgust, in which emotional suppression is associated with the activation of sympathy and has the potential for detrimental effects on health [27].

A systematic review conducted by Bria et al. [29] among health professionals in Europe identified that a high workload was correlated with high scores of emotional exhaustion and depersonalization, emphasizing that professional fulfillment is not influenced by this dimension [30]. Workload is one of the most studied predictors of burnout, both quantitatively and qualitatively, for both doctors and nurses [31,32,33,34].

The objectives of this study are represented by an evaluation of the differences between the level of burnout, workload, and organizational justice experienced by medical and non-medical staff in Romanian healthcare units, as well as examining the mediating effect of organizational justice in the relationship between workload and burnout level and the moderating effect of emotional regulation in the relationship between workload and burnout. There is no previous research on burnout, organizational justice, workload, and emotional regulation that compares medical and non-medical staff in Romania.

The hypotheses formulated for the research were the following:

**Hypothesis** **1** **(H1).** *There are significant differences in emotional exhaustion, personal accomplishment depersonalization, workload, and organizational justice between medical and non-medical staff*.

**Hypothesis** **2.1** **(H2.1).** *Organizational justice mediates the relationship between workload and emotional exhaustion, while cognitive reappraisal moderates the relationship between them*.

As reported in previously published research, workload was found to be a predictor for burnout. In the case of nurses, the lack of organizational justice is identified when procedures and organizational matters become unfair, triggering, under these conditions, emotional exhaustion and depersonalization. As cognitive reappraisal was related to personal accomplishment and low levels of burnout [31,32,33,34], a focus on the relationship between workload-cognitive reappraisal and burnout for two different categories of personnel who work in the same kind of institutions but accomplish totally different tasks was identified as being necessary.

**Hypothesis** **2.2** **(H2.2).** *Organizational justice mediates the relationship between workload and depersonalization, while expressive suppression moderates the relationship between them.*

As was mentioned earlier, expressive suppression was correlated with depersonalization. The results emphasized, in the scientific literature in the field of burnout, that in the case of healthcare personnel, emotional suppression can lead to depersonalization.

**Hypothesis** **3** **(H3).** *Cognitive reappraisal moderates the relationship between workload and depersonalization.*

Since cognitive reappraisal strategies were correlated with lower levels of burnout [31,32,33,34], we considered a model that could mitigate the levels of burnout felt when workload registered high levels, in the case of a medical and non-medical sample.

## 2. Materials and Methods

### 2.1. Study Design and Population

A cross-sectional study using the snowball sampling method was conducted between 15 October and 30 November 2022. An online questionnaire was constructed for this research; it was distributed with the help of the Order of General Nurses, Midwives, and Nurses of Romania, the Association of Legal Advisers in Public Hospitals in Romania, and the Association of Quality Management Officers in Public Hospitals. In addition, the questionnaire was distributed in private and public university hospitals in Iasi, Constanta, Oradea, Cluj, Bucharest, and Timisoara, the main university cities.

The inclusion criterion was that participants should be medical and non-medical staff currently employed in hospitals, clinics, and other public or private institutions in Romania. The exclusion criterion was that respondents filled in the questionnaires after the deadline.

The questionnaire informed the participants about the purpose of the study and that the collected data would be used only for scientific purposes. Respondents were also informed that they could withdraw from the study at any time, without consequences. No incentives were given for completing the questionnaires. Participation in the current study was voluntary and the participants’ anonymity was assured. If participants agreed to this information, the questionnaire stated that their continued completion of the questionnaire signified their agreement to participate in the study.

### 2.2. Sample Size Calculation

To calculate the required number of participants, a power analysis was performed using GPower software (version 3.1.9.2, Heinrich Heine University of Dusseldorf, Germany) [35] for the direct effects in the moderated mediation analysis, these being hierarchical regressions. For indirect effects, no problems were presented in the case of power, as these were calculated using the bootstrapping technique. In the case of a moderated mediation analysis with 1 mediator and 1 moderator at a recommended power of 1 − β = 0.80 [36], a minimum of 125 participants were required to detect an average effect (f 2 = 0.10). The final sample was composed of 230 individuals, divided into two subsamples according to the specialty of the participants (medical vs. non-medical fields). Of these, 139 held the position of medical assistant (60%; M years = 44.67) and 91 held the position of administrative staff (40%; M years = 44.31).

### 2.3. Study Instruments

The questionnaire was constructed using the Google Forms application (Alphabet, Mountain View, CA, USA). The questionnaire had two sections:

(a) The first section collected socio-demographic and medical data, along with information about the type of institution, activity, length of experience in the field, and number of years at the current workplace.
(b) The second section used psychological instruments to measure the level of burnout, workload, organizational justice, and emotional regulation.

*Burnout*—The 22-item Maslach MBI human services survey (MBI-HSS) measuring instrument [37] was used to measure the level of burnout. MBI-HSS is the original and most widely used variant of the tool. The instrument contains 22 items, to which participants are asked to respond on a Likert scale from 1 to 7, where 1 = never and 7 = always. The instrument contains three dimensions: emotional exhaustion, depersonalization, and personal accomplishment. Examples of scale items are *I feel frustrated with my work* (emotional exhaustion); *I feel that I influence other people positively through my work* (personal accomplishment); and *I’m not really interested in what is going on with many of my colleagues* (depersonalization). High scores for emotional exhaustion and depersonalization dimensions and low scores for personal accomplishment correspond to higher levels of burnout. Burnout severity is classified according to the subscales, with a high score of emotional exhaustion and depersonalization (low score of personal accomplishment) (low: 1/3 dimension, moderate: 2/3 dimensions, and severe: 3/3 dimensions) [4,24,37,38,39]. In the research sample, Cronbach’s alpha consistency coefficients for each subscale range from acceptable to very good, namely, α = 0.93 (emotional exhaustion), α = 0.75 (personal accomplishment), and α = 0.71 (depersonalization).

*Workload and organizational justice*—the ECO system [38] was used for measuring overload and organizational justice, via the dimensions of workload (8 items) and organizational justice (8 items) to measure the psychosocial risks at the organizational level. It was measured on a 5-point Likert scale, where 1 = to a very small extent and 5 = to a very large extent. The scale was used in the past in organizations in Romania; it has its own specificity, where employees are under the influence of similar professional pressures and working conditions but hold different positions in an organization, as in our study (medical and non-medical staff). This dimension of workload presented adequate psychometric properties in previous studies (Cronbach’s alpha > 0.78) and organizational justice (Cronbach’s alpha > 0.81) and in the present one [39]. For the research sample, a very good internal consistency coefficient was found for the workload sub-scale, α = 0.90. On the research sample, an acceptable internal consistency coefficient was found for the organizational justice sub-scale, α = 0.77.

*Emotional regulation*—The emotion regulation questionnaire [40] was used to measure emotional regulation. The items assess two emotional regulation strategies: expressive suppression (4 items) and cognitive reappraisal (6 items). Each item is measured on a 7-point Likert scale from 1 to 7, where 1 = totally disagree, and 7 = totally agree. The tool was also used in previous studies in the medical field [41]. On the research sample, a good internal consistency coefficient is found for the cognitive reappraisal sub-scale, α = 0.83. On the research sample, a good internal consistency coefficient was found for the expressive suppression subscale, α = 0.85.

The dependent variables considered for the present research were emotional exhaustion, depersonalization, and personal accomplishment. The independent variable taken into consideration was workload and organizational justice was considered as the mediating variable. Expressive suppression and cognitive reappraisal were considered moderating variables.

### 2.4. Statistical Analysis

All analyses for this research were performed using the IBM Statistical Package for Social Sciences (SPSS) Statistics 229 for Windows, version 29 (SPSS Inc., Chicago, IL, USA) for hypothesis testing and the statistical analysis of the data, then using the process extension [42] to perform the moderated mediation analysis. The participants were divided into 2 sub-samples, according to their field of activity within the medical units, namely, medical assistants (medical field) or administrative staff (non-medical field), to be able to observe possible differences between these 2 groups.

### 2.5. Ethical Statement

The study was conducted in accordance with the Declaration of Helsinki and approved by the Institutional Review Board of the Association of Legal Advisors from Public Hospitals in Romania, Research Ethical Agreement No. 54/15, September 2022.

## 3. Results

### 3.1. Socio-Demographic and Job-Related Data

The sample consisted of 139 nurses and 91 administrative staff respondents. Most participants were female (94.3%), with an age of about M = 44.67 ± 8.19 for medical staff and M = 44.31 ± 9.30 for non-medical staff.

The majority of them (85.7) were in a relationship, and 81.7% declared that they had at least one child. More than a quarter of them (26.5%) reported that they suffered from a chronic disease.

Approximately 40% had a bachelor’s degree. Regarding their experience in the field, we identified that 41.7% had completed at least 20 years of employment, 87% of respondents had more than one job, 83.9% were working in a public institution, and 33.5% were working in an emergency unit.

Almost one-fifth of the respondents declared that they had a managerial position. Detailed socio-demographic data are presented in Table 1.

### 3.2. MBI, Organizational Justice and Emotion Regulation

As expected, significant, strong correlations between overload and emotional exhaustion can be observed across both work domains (r = 0.76; *p <* 0.01). In the case of those working in the medical field, another strong, negative correlation exists, this time between organizational justice and emotional exhaustion (r = −0.49; *p* < 0.01), indicating that when the sense of justice in the workplace is higher, emotional exhaustion is lower and vice versa.

MBI Dimensions Scores: *Medical personnel*—Overall, scores for emotional exhaustion for medical personnel are high; depersonalization is moderate and personal accomplishment is high. The study sample mean scores for the emotional exhaustion, depersonalization, and personal accomplishment subscales were 27.05, 8.26, and 47.35, respectively. Regarding *non-medical personnel,* overall, scores for emotional exhaustion for non-medical personnel were proven to be high; depersonalization was moderate and personal accomplishment was high. The study sample mean scores for the emotional exhaustion, depersonalization, and personal accomplishment subscales were 35.84, 11.79, and 44, respectively. The mean scores for emotional exhaustion indicate a high level for this dimension for both medical and non-medical personnel, along with moderate scores for depersonalization for both medical and nonmedical staff. However, the personal accomplishment mean score indicated a high level of personal accomplishment in both the subcategories of participants.

*Workload Dimension*—Mean scores for workload dimensions were M = 20.35 for medical personnel and M = 25.34 for non-medical personnel, pointing to higher overload for non-medical personnel.

*Organizational justice*—Mean scores for organizational justice dimensions were 25.87 for medical personnel and 21.34 for non-medical personnel, pointing to higher organizational justice for medical personnel.

*Emotional regulation dimensions*—Mean scores for cognitive reappraisal were M = 32.02 for medical personnel and M = 31.67 for non-medical personnel, suggesting that medical personnel are relying on more cognitive reappraisal regulation. Mean scores for the other dimension of emotion regulation, expressive suppression, was M = 17.99 for medical personnel and M = 17.19 for non-medical personnel. Appropriate scores were registered for both categories of personnel but showed higher scores for both dimensions of emotional regulation in the case of medical personnel.

More detailed results and the correlation analysis are presented in Table 2.

### 3.3. Inferential Data Analysis

**Hypothesis** **1** **(H1).** *There are significant differences in emotional exhaustion, personal accomplishment depersonalization, workload, and organizational justice between medical and non-medical staff*.

To test this hypothesis, an independent samples *t*-test was used. Thus, the results indicate that there are significant differences between the two groups in terms of emotional exhaustion (*p* < 0.001), personal accomplishment (*p* < 0.001), depersonalization (*p* < 0.001), workload (*p* < 0.001), and organizational justice (*p* < 0.001). Medical participants are significantly more professionally fulfilled and experience organizational justice significantly more strongly, while administrative staff experience significantly more emotional exhaustion, depersonalization, and workload.

**Hypothesis** **2.1** **(H2.1).** *Organizational justice mediates the relationship between workload and emotional exhaustion, while cognitive reappraisal moderates the relationship between them*.

**Figure 1:**
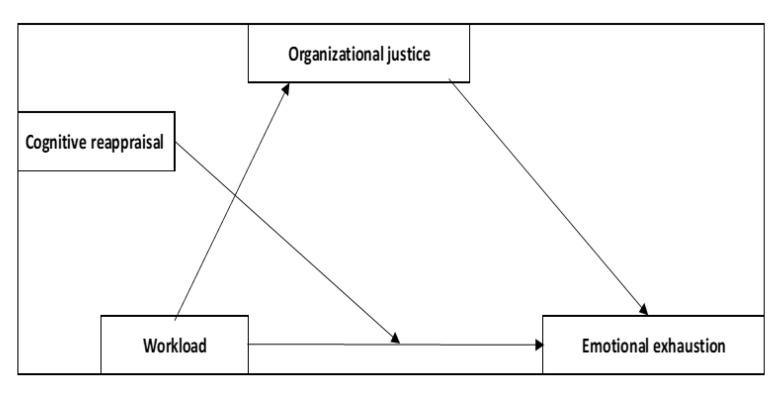
Model 1.

Thus, a high workload leads to a low sense of organizational justice, and these, together, affect emotional exhaustion. Cognitive reappraisal moderates the relationship by interacting with overload, with the latter changing its effect strength as a function of cognitive reappraisal values. The moderated mediation model—Model 1 (see Figure 1)—was applied separately to the medical staff and then to the non-medical staff. For this hypothesis, moderated mediation analysis was applied using process extension [42].

For nurses, a significant direct effect of workload on organizational justice (path a1; b = −0.30, SE = 0.06, *p* < 0.001) was found. The effect of organizational justice on emotional exhaustion (path b1) was also significant (b = −0.51, SE = 0.11, *p* < 0.001). The direct effect of workload on emotional exhaustion (pathway c) was insignificant (b = −0.93, SE = 0.47, *p* > 0.05) but, in contrast, the indirect effect through organizational justice (pathway a1b1) was significant (b = 0.15, SE = 0.05, 95% CI [0.05; 0.27]), indicating a total mediation.

Finally, the total effect (pathway c’) was significant (b = 0.18, SE = 0.07, 95% CI [0.05; 0.33]). The interaction between cognitive reappraisal and workload was not a significant one (b = 0.00, SE = 0.01; *p* > 0.05), therefore the former was not a significant moderator. Thus, a sense of justice at work was a significant mediator in the mediation model. These results indicate that a high level of workload corresponds to a low level of justice, and ultimately, emotional exhaustion is felt more strongly. The mediation model explained 63% of emotional exhaustion (R^2^ = 0.63, *p* < 0.05).

For non-medical staff, workload had a significant direct effect on organizational justice (path a; b = −0.17, SE = 0.07, *p* < 0.05). All effects on emotional exhaustion were nonsignificant (*p* > 0.05), as was the interaction effect between reassessment and overload. Thus, for non-medical staff, the pattern is insignificant.

**Hypothesis** **2.2** **(H2.2).** *Organizational justice mediates the relationship between workload and depersonalization, while expressive suppression moderates the relationship between them*.

**Figure 2:**
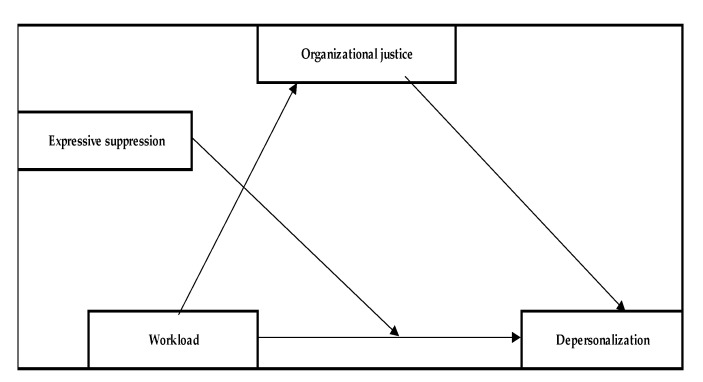
Model 2.

For medical staff, a significant direct effect of workload on justice (path a; b = −0.30, SE = 0.06, *p* < 0.001) was found, as well as on depersonalization (path c; b = 0.45, SE = 0.13, *p* < 0.001). However, the effect of justice on depersonalization (pathway b) was insignificant (b = 0.00, SE = 0.05, *p* > 0.05), whereas the interaction between workload and expressive suppression was significant (b = −0.01, SE = 0.00, *p* < 0.05), resulting in significant moderation. The moderation model—Model 2 (see Figure 2)—explained 14% of the depersonalization (R_2_ = 0.14, *p* < 0.001), while the Johnson–Neyman significance values indicated that the effect of workload on depersonalization becomes insignificant at the 60th percentile of emotional suppression scores. Thus, it appears that at low (−1SD) or medium suppression scores, the effect of workload on depersonalization is significant and positive, whereas, at high suppression scores (+1SD), a high workload does not result in high depersonalization, and the effect is insignificant.

For non-medical staff, workload had a significant direct effect on organizational justice (path a; b = −0.17, SE = 0.07, *p* < 0.05), but no significant direct effect on depersonalization (path c; b = 0.11, SE = 0.23, *p* > 0.05). Although the effect of justice on depersonalization (pathway b) was significant (b = −0.21, SE = 0.10, *p* < 0.05), the indirect effect was non-significant (b = 0.03, SE = 0.02, 95% CI [−0.00; 0.10]), as was that of moderator interaction, resulting in a non-significant moderated mediation model.

**Hypothesis** **3** **(H3).** *Cognitive reappraisal moderates the relationship between workload and depersonalization*.

**Figure 3:**
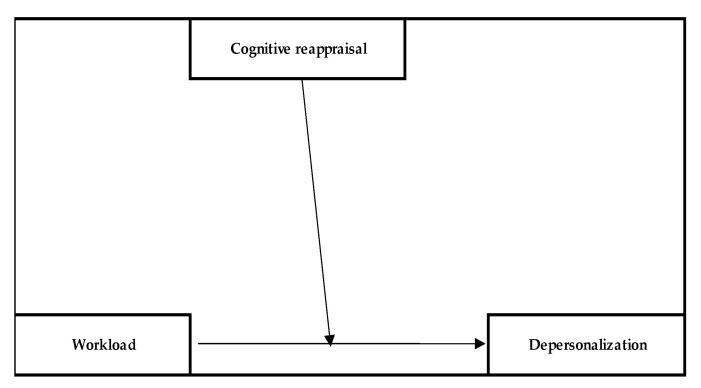
Model 3.

For nurses, a significant effect of workload on depersonalization was observed (path c; b = 0.1.10, SE = 0.21, *p* < 0.001) as was the moderator interaction effect (b = −0.03; SE = 0.00, *p* < 0.001). Thus, the moderation effect was significant, resulting in a moderation model explaining 23% of the variance in depersonalization (R_2_ = 0.23, *p* < 0.001) (Model 3 (see Figure 3)).

It was observed that at low (−1SD) levels of reappraisals, the effect of workload on depersonalization was significant and positive (b = 0.29; SE = 0.05, *p* < 0.001), whereas, at medium levels of reappraisals, the effect decreased (b = 0.15; SE = 0.03, *p* < 0.001) but remained significant. In contrast, at high levels of reappraisals (+1SD), the effect became insignificant (b = −0.01; SE = 0.05, *p* > 0.05). Finally, the Johnson–Neyman significance values tell us that at the 63rd percentile of reappraisal scores, the effect of overload on depersonalization became insignificant, and at the 97th percentile, it became significant again but changed valence and became negative.

In contrast, among non-medical staff, all effects on depersonalization were insignificant (*p* > 0.05), indicating that the moderation model was not significant for this subsample.

## 4. Discussion

Medical staff scored high rates for the emotional exhaustion dimension, as well as non-medical staff. For the depersonalization dimension, moderate scores were recorded for both medical and non-medical staff. Thus, the results are in line with previous studies that found comparative values for these dimensions in health professionals [43]. Surprisingly, both medical and non-medical staff scored highly on the personal accomplishment dimension. Thus, although employees feel emotionally drained and depersonalized, they have high feelings of fulfillment at work. Similar results have been obtained in other healthcare professionals [44], thus revealing that despite the emotional exhaustion and depersonalization that is felt, professional fulfillment can arise from the positive impact of treating patients, in the case of nurses, and facilitating the medical act, in the case of non-medical staff.

Another study reveals that 15.38% of physicians, 18.18% of nurses, and 10% of administrative staff met the criteria for a high level of burnout [45]. The role of administrative staff is well defined in the medical field and they frequently face a high level of duties, as demonstrated in a study conducted among medical staff in six hospitals in Sibiu County. The results of this research highlighted that administrative staff recorded high scores for burnout dimensions since this category of staff does not work in shifts, being forced to complete their tasks while working at a comparatively low salary level compared to other health professionals [46]. Regarding personal accomplishment, the findings of the present study identified that nurses are more professionally fulfilled than administrative staff; this could be interpreted by the fact that nurses feel very energetic and have achieved many valuable goals in their work.

Regarding the lower level of organizational justice felt by non-medical staff, we can assess that this injustice could be caused by the salary inequalities between the two categories of staff. In terms of the current research, we can appreciate that unlike medical staff, who have benefitted from a higher salaried income since 2018, according to the Law on the Salary of Public Fund Staff, number 153/2017 [47], administrative staff will receive a much lower monthly income. Moreover, during the COVID-19 pandemic, medical staff received bonuses of between 75% and 85% for the challenging nature of medical work during that period, while administrative staff worked under the same financial conditions as before. We can say that the lower level of organizational justice felt by this category of staff could also be due to this unique salary law, which has created an unequal pay ratio for different categories of specialists working in healthcare units. Given that the present study was conducted shortly after the end of the pandemic, the high level of workload, depersonalization, and emotional exhaustion experienced could also be attributed to the post-COVID-19 period. Finally, if we consider the COVID-19 period, we can appreciate that administrative staff had to carry out their work from their workplace and not in an online environment, as with other administrative employees in corporations, institutions, and other companies that adopted the telework system [48]. This sense of injustice could also be caused by the constantly changing legislation, a high workload, and administrative staff being faced with the constant drafting, modification, and evaluation carried out for the smooth running of work in hospital units. Under these conditions, non-medical staff feel depersonalized, become impersonal in their relations with their interlocutors, are harsher, and, finally, may feel indifferent regarding their relationships with others.

The lower perceived level of organizational justice for non-medical staff, as well as a high level of workload dimension for this category of staff, could also be explained by the assigning of numerous tasks with short deadlines. Previous research has shown that one of the factors leading to a high level of burnout is a high level of workload caused by overload, which occurs in the work environment when a high level of job stress is experienced repeatedly. Regarding administrative staff, the overload and the level of emotional exhaustion could be explained based on a high workload, hours spent working overtime, and a decreased level of organizational justice being recorded [14]. Given that administrative staff experience a higher level of emotional exhaustion and overload, the results can be attributed to differences in the various duties of the two categories of staff, as well as differences between the organization of the medical department and the administrative one.

Given that medical participants are more professionally fulfilled and experience significantly higher organizational justice, while administrative staff experience significantly more emotional exhaustion, depersonalization, and overload, we can say that this finding is congruent with those of Maslach et al. [4], who found that a sense of inequity could ultimately lead to burnout. In addition, in light of resource conservation theory [49], resource depletion over time may be a stressor that ultimately leads to burnout [50]. This supports the idea that there may be a direct, logical, and proven link between organizational justice and burnout. Practically, the organization should be aware of the importance of organizational justice perceptions among its employees, their implications in accelerating the burnout process (at all levels of the organization), and the impact on its operations [37]. Organizational justice, described as treating all staff ethically, involves the fair allocation of tasks, as well as strategies and methods to treat individuals fairly in the workplace [51].

For those working in the medical sector, another strong correlation, this time negative, is seen between organizational justice and emotional exhaustion, indicating that when the sense of justice in the workplace is higher, emotional exhaustion is lower, and vice versa. The results thus reported appear when introducing organizational justice as a mediator in the relationship between overload and emotional exhaustion. The dimension of justice alone is stronger in non-medical staff, probably because medical personnel have other resources with which to register greater organizational justice and because their work saves lives.

The present study clarified that there was a significant relationship between organizational justice and the level of burnout experienced at work. This result is consistent with previous findings demonstrating that feelings of injustice can occur when hospital professionals find procedures, policies, or interactions unfair and, thus, become emotionally exhausted, depersonalized, and experience a lack of professional fulfillment [11].

Regarding the mediation model between workload, organizational justice, and emotional exhaustion, in the case of nurses, the level of perceived organizational justice is directly affected by workload. The effect of organizational justice on emotional exhaustion is also significant but the direct effect of workload on emotional exhaustion is insignificant; instead, the indirect effect via organizational justice is significant and indicates full mediation. Thus, the results indicate that a high level of workload corresponds to a low level of organizational justice and, ultimately, emotional exhaustion is felt more strongly; thus, the mediation model involving organizational justice explains 63% of the emotional exhaustion. Perceptions of unfairness can threaten employees’ well-being and give them an inappropriate sense of reward for investing personal resources, they can become frustrated and even become worn out. As other authors have highlighted, if employees experience burnout and disequilibrium (due to injustice, in this case), they are likely to aspire to regain and maintain their emotional balance [52].

The mediation model was also not significant in the case of non-medical staff, although a significant direct effect of workload on organizational justice was found. In the case of non-medical personnel, even if workload (numerous duties, overtime, or working without interruption) has a significant effect on attitudes to organizational justice, the connection affects the way administrative staff feel about their professional competency, remuneration, or task distribution. Hence, the perception of organizational justice felt by respondents is not enough to prevent feelings of emotional exhaustion. Thus, even if participants feel that they are fairly treated by superiors and that their performance is valued, if they must complete too many duties, work harder than their colleagues, and engage in overtime, they will feel emotional exhaustion. Additionally, the moderating model by means of cognitive reappraisal, regarding the relationship between emotional overload and exhaustion, did not prove significant, either in the case of medical personnel or in the case of non-medical personnel. Facing a problem and having a positive attitude are not enough to prevent feelings of emotional exhaustion from being determined by overload. These results are consistent with those reported in other research, which has not demonstrated a link between the habitual use of reappraisal and levels of burnout [53]. In the case of nurses, workload produces its effects on emotional exhaustion not directly but indirectly, through organizational justice. That is, at low levels of organizational justice and high levels of workload, nurses experience high levels of emotional exhaustion, but in the absence of justice, workload no longer has a direct effect on emotional exhaustion. Thus, considering the level of emotional exhaustion experienced by nurses, we can appreciate that this finding is in line with previous studies that registered high levels of burnout dimensions, which could be attributed to the complexity of the quality of care and the high level of work volume, dimension workload, and lack of organizational justice, as well as poor management [11].

In the case of the mediation model between workload, organizational justice, and depersonalization for medical personnel, it was highlighted that a high level of workload can lead to feeling burnout through the depersonalization dimension. Overload has a direct effect on perceptions of organizational justice, as does depersonalization, but the mediating effect is insignificant. Under these conditions, organizational justice did not prove to be a significant mediator in the relationship between overload and depersonalization. Thus, we can say that regardless of the levels of organizational justice experienced by nurses, they can feel depersonalized in their relationship with the patient when they feel overwhelmed by the tasks they must complete. This is congruent with previous studies that correlated depersonalization with nursing insensitivity developed over time, after nurses had accumulated seniority in their specialty and experienced a high level of workload [11]. In the case of non-medical staff, workload has a significant direct effect on organizational justice but not a significant direct effect on depersonalization. Although the effect of justice on depersonalization is significant, the indirect effect is insignificant and, thus, the model is insignificant. In this case, the mediation model was non-significant for both medical staff and non-medical staff. Thus, the workload felt by respondents, such as overtime or the volume of tasks, results in depersonalization. Hence, if the respondents are dealing directly with patients, or even if they work in the administrative unit, they will have feelings of detachment within the network of patients, colleagues, or supervisors. Even if they feel that they are treated fairly by the organization or by their superiors, workload will result in a feeling of detachment from their jobs.

The moderating model, workload–emotional suppression–depersonalization, was significant only in the case of medical personnel. Medical personnel are trained to deal with negative emotions, develop positive relationships with patients, control their emotions, and detach themselves from negative events. This lack of positive or negative emotions could reduce the level of depersonalization only in the case of medical personnel. In the case of non-medical personnel, the need to control emotions is less learned and practiced, and it may not be enough to alleviate feelings of detachment. Therefore, when overwork is significant, emotional regulation is not sufficient to mitigate the levels of depersonalization experienced by administrative staff.

Previous studies showed that expressive suppression was associated with depersonalization, while cognitive reappraisal was related to personal accomplishment. These associations occurred in the expected direction; that is, expressive suppression was associated with a high level of burnout, while cognitive reappraisal strategies correlated with lower levels of burnout [41]. Emotional suppression has a moderating effect between workload and depersonalization; thus, at low or medium levels of emotional suppression, workload leads to depersonalization, while at high suppression scores, a high workload does not result in high depersonalization, the effect becoming insignificant. The results are in line with previous studies offering the finding that in the case of health professionals, the emotional suppression variable can lead to depersonalization, highlighting the fact that managing emotions is vital in the healthcare environment [41].

Considering the Johnson–Neyman values regarding the moderating effect of cognitive reappraisal in the relationship between workload and depersonalization, we can appreciate that for those with an average or below-average level of reappraisal, a high workload leads to high depersonalization; for those with an above-average level of reappraisal, overload becomes a nonsignificant predictor; in those with extremely high reappraisal, high workload leads to low depersonalization, indicating the importance of cognitive reappraisals, which may change the dynamics of the relationship between workload and depersonalization. For example, compensating for organizational inefficiencies by performing unnecessary tests/procedures creates internal conflict. Semmer et al. [54] demonstrate that illegitimate tasks predict work effort more reliably than other potential stressors, such as social stressors or perceived injustices. These results are confirmed by previous studies, which, among the cognitive emotion regulation strategies examined, found that positive reappraisal was significantly negatively associated with depersonalization [55].

Although, in the case of medical personnel, cognitive re-evaluation may matter, in the case of non-medical personnel, this is no longer valid; depersonalization takes place in non-medical personnel, regardless of the recorded cognitive re-evaluations. This contrasts with previous studies that consistently demonstrate a protective impact of cognitive reappraisal on burnout among health workers and other professionals in general [55,56]. Therefore, for administrative personnel, more positive emotions and fewer negative emotions will not reduce feelings of detachment from their colleagues or superiors.

### 4.1. Strengths and Limitations of the Study

The strength of the present study is first due to its hypothesis, verified with Romanian medical staff; second, it is due to its generalizability, considering the fact that the questionnaire was distributed in the main regions in Romania. The limitations of the research are related to the fact that some of the variables were assessed with few items because the questionnaire already had many items; measuring all of them with whole scales would result in a very long questionnaire, which would certainly discourage participants from responding. However, the internal consistency measures of the dimensions used were very good. A second limitation is related to the samples, as we had a snowball sample. The sample showed good variability in most socio-demographic and occupational variables, which supports its heterogeneity and, therefore, the generalizability of these results to similar samples. Another limitation concerns the fact that all items were self-reported, which could lead to a possible overestimation of correlations between variables.

### 4.2. Future Research Directions and Practical Implications

Future studies should continue this research to include other categories of employees in health facilities, such as doctors and auxiliary staff, to check whether the mediation–moderation model has an effect in the case of other categories of health professionals. The results of the present study could help researchers in the future to focus on variables that are strong predictors of burnout and to test models that highlight the most successful outcomes in this regard. According to our results, the underlying variables appear to be organizational justice, workload, and cognitive reappraisal.

Given that the results of the study are post-pandemic, we recommend those variables, paying particular attention to their patterns in healthcare services. However, overload should be further analyzed as a predictor of burnout, as well as emotional regulation as a protective factor against burnout. The results of the research showed that there is a need for objective feedback from both medical and non-medical staff. Constant evaluation of occupation stress and burnout, as well as identifying the level of satisfaction with one’s job, can guide and shape management staff intervention. Approaching burnout syndrome must be managed in a rigorous, scientific way, using methods such as assessment, monitoring, intervention, prevention, and paying increasing attention to vulnerable socio-professional categories. Regular consultations with the medical and non-medical staff regarding working conditions, quality of equipment, protocols, decisions, worktime, professional needs, etc., should also be considered.

## 5. Conclusions

Medical staff feel a higher level of personal accomplishment and organizational justice than non-medical staff. Similarly, non-medical staff experience a higher level of overload, emotional exhaustion, and depersonalization.

When mediators and moderators interfere, in the case of nurses, workload has a direct effect on organizational justice, which, in turn, directly affects emotional exhaustion, whereas cognitive reappraisal does not. Organizational justice was found to be a significant mediator in the relationship between workload and emotional exhaustion. Thus, the mediation model is significant, but the one concerning moderation by cognitive reappraisal in the relationship between workload and emotional exhaustion is insignificant. For non-medical staff, a significant direct effect of justice workload and justice on depersonalization was found. All of the effects regarding emotional exhaustion and the interaction between cognitive reappraisal and workload were nonsignificant.

Workload directly affects organizational justice and depersonalization in the case of medical staff, but organizational justice does not directly affect depersonalization. Thus, organizational justice did not prove to be a reliable mediator between workload and depersonalization, neither in the case of medical personnel nor in the case of non-medical personnel. It was also found that expressive suppression has a moderating effect on the relationship between overload and depersonalization in the case of medical staff. The model is also not significant in the case of non-medical personnel, although overload directly affects organizational justice, which, in turn, directly affects depersonalization in the case of this category of personnel. Finally, in the case of medical staff, it was found that cognitive reappraisal moderates the relationship between overload and depersonalization; thus, the moderating effect proved significant. It was observed that at low levels of cognitive reappraisal, the effect of overload on depersonalization is significant and positive, while at medium levels of re-evaluation, the effect decreases but remains significant, and at high levels of re-evaluation, the effect becomes insignificant.

## Figures and Tables

**Table 1:** Characteristics of the study sample (*n* = 230).

Characteristics	N	%
Gender		
Male	13	5.7%
Female	217	94.3%
Marital status		
Single	33	14.3%
In a relationship	197	85.7%
Children		
No	42	18.3%
Yes	188	81.7%
Professional category		
Nurse	139	60.4%
Administrative personnel	91	39.6%
Job tenure in years		
5 years or less	33	14.3%
Between 6 and 10 years	38	16.5%
Between 11 and 20 years	63	27.4%
More than 20 years	96	41.7%
Employment		
Single job	30	13.0%
More than one job	200	87.0%
Type of organization		
Public	193	83.9%
Private	21	9.1%
Public and private	16	7.0%
Emergency unit		
Yes	77	33.5%
No	153	66.5%
Team coordinator		
Yes	46	20.0%
No	184	80.0%
Education		
High school/post-secondary studies	82	35.7%
Certificate or qualification diploma	6	2.6%
Bachelors’ degree	92	40.0%
Master’s degree	50	21.7%
Chronic diseases		
Yes	61	26.5%
No	169	73.5%

**Table 2:** Descriptive statistics and Pearson correlations ^1^.

	M (SD)	1	2	3	4	5	6	7	8
Medical									
1. Age	44.67 (8.19)	-							
2. Emotional exhaustion	27.05 (12.34)	0.19 *	0.21 **	-					
3. Personal accomplishment	47.35 (6.78)	−0.07	−0.28 **	−0.24 **	-				
4. Depersonalization	8.26 (3.95)	0.02	0.21 *	0.29 **	−0.31 **	-			
5. Workload	20.35 (7.65)	0.16	0.22 **	0.76 **	−0.24 **	0.29 **	-		
6. Cognitive reappraisal	32.02 (5.37)	−0.12	0.09	0.05	0.13	−0.14	0.05	-	
7. Expressive suppression	17.99 (5.61)	0.10	−0.06	0.15	0.06	−0.12	0.09	0.25 **	-
8. Organizational justice	25.87 (6.02)	−0.17 *	0.03	−0.49 **	0.14	−0.09	−0.38 **	0.11	−0.12
Non-Medical									
1. Age	44.31 (9.30)	-							
2. Emotional exhaustion	35.84 (14.71)	0.23 *	0.04	-					
3. Personal accomplishment	44 (7.37)	−0.01	0.18	−0.08	-				
4. Depersonalization	11.79 (6.30)	−0.12	0.25 *	0.41 **	−0.06	-			
5. Workload	25.43 (7.87)	0.21 *	0.26 **	0.76 **	0.04	0.45 **	-		
6. Cognitive reappraisal	31.67 (6.19)	0.07	−0.04	0.16	0.28 **	−0.08	−0.03	-	
7. Expressive suppression	17.19 (5.53)	0.06	−0.09	0.07	0.14	0.00	0.07	0.06	-
8. Organizational justice	21.34 (5.72)	−0.09	0.19	−0.29 **	0.11	−0.28 **	−0.23 *	0.23 *	−0.03

^1^ N_medical_ = 139; N_non_ = 91; * *p* < 0.05; ** *p* < 0.01.

## Data Availability

Data are available upon request from the corresponding author.
